# DNA‐Directed Patterning for Versatile Validation and Characterization of a Lipid‐Based Nanoparticle Model of SARS‐CoV‐2

**DOI:** 10.1002/advs.202101166

**Published:** 2021-10-21

**Authors:** Molly Kozminsky, Thomas R. Carey, Lydia L. Sohn

**Affiliations:** ^1^ California Institute for Quantitative Biosciences University of California Berkeley 174 Stanley Hall Berkeley CA 94720 USA; ^2^ UC Berkeley–UC San Francisco Graduate Program in Bioengineering University of California, Berkeley 306 Stanley Hall Berkeley CA 94720 USA; ^3^ Department of Mechanical Engineering University of California Berkeley 5118 Etcheverry Hall Berkeley CA 94720 USA

**Keywords:** DNA‐directed patterning, liposomes, neutralizing antibodies, SARS‐CoV‐2, spike

## Abstract

Lipid‐based nanoparticles have been applied extensively in drug delivery and vaccine strategies and are finding diverse applications in the coronavirus disease 2019 (COVID‐19) pandemic—from vaccine‐component encapsulation to modeling the virus, itself. High‐throughput, highly flexible methods for characterization are of great benefit to the development of liposomes featuring surface proteins. DNA‐directed patterning is one such method that offers versatility in immobilizing and segregating lipid‐based nanoparticles for subsequent analysis. Here, oligonucleotides are selectively conjugated onto a glass substrate and then hybridized to complementary oligonucleotides tagged to liposomes, patterning them with great control and precision. The power of DNA‐directed patterning is demonstrated by characterizing a novel recapitulative lipid‐based nanoparticle model of severe acute respiratory syndrome coronavirus 2 (SARS‐CoV‐2)—S–liposomes—that presents the SARS‐CoV‐2 spike (S) protein on its surface. Patterning a mixture of S–liposomes and liposomes that display the tetraspanin CD63 to discrete regions of a substrate shows that angiotensin‐converting enzyme 2 (ACE2) specifically binds to S–liposomes. Subsequent introduction of S–liposomes to ACE2‐expressing cells tests the biological function of S–liposomes and shows agreement with DNA‐directed patterning‐based assays. Finally, multiplexed patterning of S–liposomes verifies the performance of commercially available neutralizing antibodies against the two S variants. Overall, DNA‐directed patterning enables a wide variety of custom assays for the characterization of any lipid‐based nanoparticle.

## Graphical abstract

DNA‐directed patterning of liposomes is a novel method to perform high‐throughput characterization of different populations of immobilized nanoparticles. It is demonstrated by validating a lipid‐based nanoparticle model of severe acute respiratory syndrome coronavirus 2 (SARS‐CoV‐2), spike–liposomes, through assays that measure binding of angiotensin‐converting enzyme 2 as well as the efficacy of broadly neutralizing antibodies.

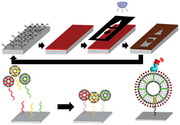

## 1 Introduction

Highly studied and widely used,^[^ ^1^ ^]^ liposomes are nanoparticles comprising a lipid bilayer membrane whose surface modifications can be used for biological targeting and accumulation.^[^ ^2^, ^3^ ^]^ Such surface modifications include targeting ligands (e.g., carbohydrates, peptides, and proteins^[^ ^1^ ^]^) that can enhance their interface with a target cell.^[^ ^4^ ^]^ For example, liposomes with envelope glycoproteins inserted into their membrane,^[^ ^3^, ^5^ ^]^ termed “virosomes,” have been utilized in Epaxal (hepatitis A) and Inflexal V (influenza) vaccines.^[^ ^5^ ^]^ Within the context of the coronavirus disease 2019 (COVID‐19) pandemic, liposomes have found important applications. Most notably, they encapsulate messenger ribonucleic acid (mRNA) in the Moderna and Pfizer‐BioNTech^[^ ^6^ ^]^ COVID‐19 vaccines. As well, they have also been functionalized with angiotensin‐converting enzyme 2 (ACE2) or neutralizing antibodies as a strategy to clear severe acute respiratory syndrome coronavirus 2 (SARS‐CoV‐2),^[^ ^7^ ^]^ the enveloped coronavirus responsible for COVID‐19. Because enveloped viruses have been shown to fuse with liposomes,^[^ ^8^ ^]^ lipid‐based nanoparticles have been suggested as models of enveloped viruses, themselves.^[^ ^9^, ^10^ ^]^ Based on their ability to display surface antigens, liposomes have been used to model immune response to antigens^[^ ^11^ ^]^ and to trigger a T‐cell response to SARS‐CoV.^[^ ^12^ ^]^ Overall, as liposome applications increase and their surface modifications grow more sophisticated, lipid‐based nanoparticle characterization is critical.

Common methods used to characterize lipid‐based nanoparticles focus on the structure of the liposomes,^[^ ^13^ ^]^ such as dynamic light scattering or X‐ray scattering. Other methods, such as polyacrylamide gel electrophoresis and subsequent staining or adapted flow cytometry,^[^ ^14^ ^]^ focus on the presence, rather than functionality, of the surface proteins. Microscopy techniques,^[^ ^15^ ^]^ such as cryo‐electron microscopy and freeze‐fracture electron microscopy, have exceedingly low throughput and require the fixation and destruction of the sample. In the field of liposome development, there is a clear need for high‐throughput, flexible and customizable, nondestructive methods that functionally assess proteins on the liposome surface.

We have developed a novel, high‐throughput method, DNA‐directed patterning,^[^ ^16^ ^]^ to validate lipid‐based nanoparticles. DNA‐directed patterning relies on lithographically patterning single‐stranded oligonucleotides on a glass substrate and subsequently hybridizing the complementary oligonucleotides to which liposomes have been tagged, ultimately leading to the controlled and precise immobilization of liposomes. While DNA–liposome conjugates have previously been used as biosensors or components in DNA microarrays,^[^ ^17^, ^18^, ^19^ ^]^ here, we use DNA to specifically pattern liposomes to perform a variety of assays that rapidly characterize them. We demonstrate the power of our method through the development of S–liposomes—a convenient, safe model of SARS‐CoV‐2 that preserves its most basic features and function, i.e., a lipid bilayer nanoparticle that displays the SARS‐CoV‐2 spike (S) protein. We show that ACE2 specifically binds to patterned arrays of S–liposomes. We subsequently confirmed S–liposome–ACE2 binding through a cell‐culture‐based assay using ACE2‐expressing cells. Finally, we also show that we can efficiently and precisely measure the ability of neutralizing antibodies to interfere with this interaction. In general, the flexibility, speed, and minimal sample preparation that high‐throughput DNA‐directed patterning affords can significantly advance novel assays focused on characterizing lipid‐based nanoparticles.

## 2 Main Text

We fabricated S–liposomes by rehydrating and extruding thin films, which include a lipid conjugated to a Ni–nitrilotriacetic acid complex, and subsequently incubating the resulting liposomes with His‐tagged spike (see the Experimental Section). Using nanoparticle tracking analysis, we determined that freshly extruded liposomes before labeling were 127.4 +/− 31.8 nm in diameter; after labeling with the spike trimer, they were 190.3 +/− 45.1 nm (Figure S1 and Table S1, Supporting Information). These diameters are comparable to that of SARS‐CoV‐2.^[^ ^20^, ^21^ ^]^

**Figure 1:**
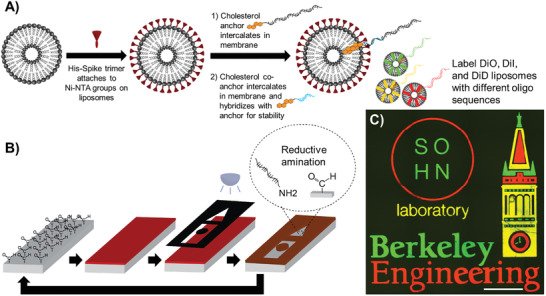
Fabrication and high resolution, spatially complex DNA‐directed patterning of liposomes. A) Liposomes are labeled with the SARS‐CoV‐2 spike protein and tagged with single‐stranded (ss) oligonucleotides conjugated to a cholesterol molecule (orange) that intercalates into the lipid bilayer. B) DNA‐directed patterning of liposomes is first accomplished by patterning ss oligonucleotides using traditional photolithography. Photoresist is spin‐coated and baked on aldehyde‐coated substrates, patterned with a photomask using UV light, and then developed. Reductive amination of exposed aldehyde groups on the patterned substrates with amine‐terminated ss oligonucleotides is subsequently performed. Photoresist is stripped with acetone for additional patterning of orthogonal‐sequence ss oligonucleotides. C) Hybridization of oligonucleotide tags on the liposomes with complementary oligonucleotides patterned onto substrates yields intricate and high‐spatial resolution patterns. Scale bar = 500 µm.

Following the fabrication of S–liposomes, we utilized DNA‐directed patterning to validate our ability to fluorescently label and visualize these lipid nanoparticles. Using photolithography, we patterned 20‐nucleotide single‐stranded oligonucleotides onto distinct regions of a glass substrate (see the Experimental Section). We then tagged liposomes with complementary single‐stranded oligonucleotides conjugated to cholesterol molecules that intercalated into their lipid bilayer. As shown in **Figure** **1**A, we successfully tagged three different groups of liposomes, each featuring a different carbocyanine dye, with a unique oligonucleotide sequence. After tagging, we mixed the three labeled lipid‐based nanoparticles and flowed the mixture across the prepatterned substrate. The complementary oligonucleotide tags hybridized to the patterned oligonucleotides, leading to distinctive patterns of labeled liposomes (Figure 1B,C). Thus, through DNA‐directed patterning, we can achieve fine‐tuned and high‐spatial resolution control over liposome localization, which in turn enables us to visualize liposomes easily using conventional fluorescence microscopy. While we demonstrate a feature size of 10 µm here, sub‐micrometer features are possible using lithographic methods beyond photolithography, e.g., electron‐beam lithography.

To demonstrate the power of DNA‐directed patterning for lipid nanoparticle characterization assays, we engineered S–liposomes to display the S protein in the appropriate configuration. This protein is homotrimeric^[^ ^22^ ^]^ and consists of a S1 subunit, which contains the receptor binding domain (RBD), and a S2 subunit, which facilitates fusion and internalization.^[^ ^23^, ^24^ ^]^ The S protein RBD binds to ACE2, and the importance of this binding to the virulence of SARS‐CoV‐2 necessitated its incorporation into our model system.

**Figure 2:**
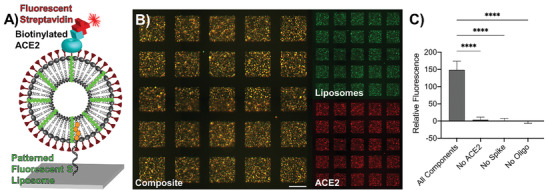
Testing ACE2 binding of S–liposomes using DNA‐directed patterning. A) Schematic representation of DNA‐directed immobilization of fluorescent liposomes displaying spike protein (S–liposomes) that bind directly to ACE2. The ACE2 is biotinylated in order to visualize it using Cy5‐conjugated streptavidin. B) Microscopy image of patterned DiO‐labeled S–liposomes and biotinylated ACE2/Cy5–streptavidin. Scale bar = 100 µm. C) Fluorescence levels corresponding to Cy5–streptavidin detection of biotinylated ACE2 with background subtracted. No fluorescence is detected when any single component of the conjugation is absent. Error bars represent standard deviation; *n* = 25 squares. ****: *p* < 0.0001, one‐way ANOVA with Tukey's multiple comparisons test.

To verify that the S trimer was oriented correctly on the liposome surface using DNA‐directed patterning, we patterned fluorescent S–liposomes onto a glass substrate in a 50 × 50 array of squares, each 141 µm × 141 µm in size. We then incubated the patterned substrate with biotinylated ACE2, which subsequently bound to the S protein. After washing the substrate thoroughly with phosphate buffered saline (PBS), we added Cy5‐conjugated streptavidin to detect the ACE2 immobilized on the S–liposomes (**Figure** **2**A). We confirmed the colocalization of streptavidin with the liposomes using fluorescent imaging (Figure 2B). To verify binding specificity, we measured fluorescence for conditions with intermediate components removed (i.e., no oligonucleotide tag, liposomes without the S protein, no addition of biotinylated ACE2; Figure 2C and Figure S2 (Supporting Information)). As expected, the condition which included all components showed significantly higher fluorescence (*p* < 0.0001, one‐way analysis of variance (ANOVA) with Tukey's multiple comparison test) than those of the controls in which each component of the binding chemistry was systematically removed. Thus, DNA‐directed patterning enabled us to confirm easily that the S trimer is oriented correctly on the S–liposomes and to measure relative spike–ACE2 binding using a straightforward fluorescence assay. In an additional show of characterization, we used DNA‐directed patterning to determine easily the stability of the liposomes up to 15 days post‐extrusion (Figure S3, Supporting Information).

**Figure 3:**
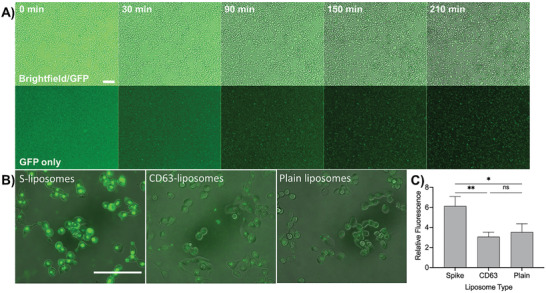
Spike–liposome binding and internalization in cells. A) Time‐lapse images show diffuse fluorescence upon the addition of DiO‐labeled S–liposomes to plated ACE2‐expressing 293T cells, then condensed, cellularly localized fluorescence as the liposomes attach to the membrane and/or are internalized. DiO liposomes were imaged using a green fluorescent protein (GFP) filter, magnification 10×, scale bar = 100 µm. B) Higher magnification image of cells shows high levels of subcellular localization of internalized spike–liposomes, in contrast to DiO CD63 or plain liposomes. Magnification 40×, scale bar = 100 µm. C) Liposomes with or without spike trimer (i.e., with no surface protein or with CD63) were added to 293T+ACE2 cells. After a 3.5 h incubation, cells were washed 3 times to remove unbound liposomes, and fluorescence was measured. S–liposomes resulted in significantly higher fluorescence intensity than unlabeled liposomes or CD63–liposomes. Error bars represent standard deviation; *n* = 3 wells for each condition. ns: not significant, *: *p* < 0.05, **: *p* < 0.01, one‐way ANOVA with Tukey's multiple comparisons test.

To validate S–liposomes further and to confirm our DNA‐directed patterning results, we investigated the ability of ACE2‐expressing cells to bind to these lipid nanoparticles. We seeded cells transduced to express ACE2 (293T+ACE2)^[^ ^25^ ^]^ and allowed them to settle overnight in a 96‐well plate, after which fluorescent liposomes, without surface protein (plain) or with S protein or CD63, a tetraspanin which is commonly found on extracellular vesicles,^[^ ^26^ ^]^ were added to the cells. To visualize liposome interaction with cells, we subsequently imaged every 30 min (**Figure** **3**A). When fluorescent liposomes were initially added to the cells, fluorescence was highly diffuse. As time elapsed, fluorescence became increasingly punctated, especially for those cells incubated with S–liposomes, indicating that liposomes were localizing to the cells. Inspection of cells under higher magnification verified that in addition to localizing on the cell membrane, liposomes were being internalized within the cells. This is in contrast to CD63 or plain liposomes, which showed far lower levels of surface localization and internalization (Figure 3B). After incubating for 3.5 h, we washed the unbound liposomes from the cells and quantified the fluorescence via a plate reader. Although some spontaneous internalization did occur with plain or CD63 liposomes, cells internalized S–liposomes at a significantly higher level (Figure 3C), in agreement with our fluorescence images. We additionally quantified fluorescence over time through the analysis of time‐lapse images to obtain uptake curves (Figure S4A, Supporting Information) and demonstrated the effect of neutralizing antibody on the binding between the S trimer presented by the liposomes and the ACE2 on the surface of the cells (Figure S4B, Supporting Information). Their interaction with ACE2‐expressing cells demonstrates that S–liposomes can serve as a model of SARS‐CoV‐2 and its variants for functional assays. Moreover, the concordance between the cellular ACE2–S–liposome binding and the DNA‐directed patterning fluorescence assays supports the broad applicability of DNA‐directed liposome patterning.

**Figure 4:**
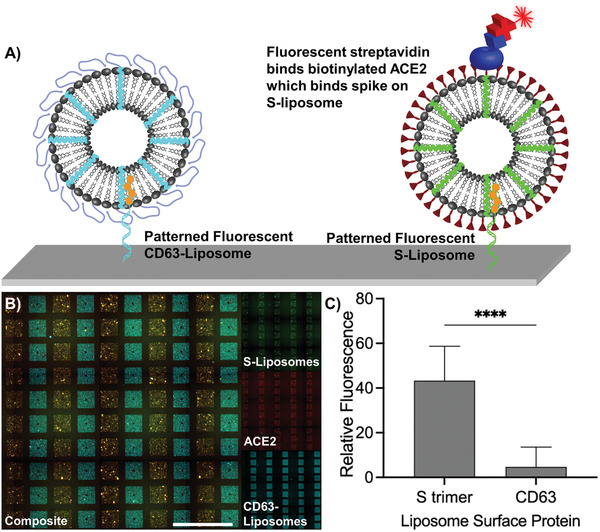
Selective binding of ACE2 to S–liposomes in the presence of liposomes presenting different surface proteins as visualized using DNA‐directed patterning. A) Schematic of S–liposomes and CD63–liposomes fluorescently labeled with different carbocyanine dyes. Based on the display of the S protein, biotinylated ACE2 followed by fluorescent streptavidin is localized only to regions of the substrate patterned with S–liposomes. B) S–liposomes (green) and CD63–liposomes (cyan) were patterned in alternating columns of an array of 141 µm by 141 µm squares, after which biotinylated ACE2 and fluorescent streptavidin were introduced. Scale bar = 500 µm. C) Fluorescence associated with Cy5–streptavidin is significantly higher in regions of the substrate patterned with liposomes displaying the S trimer than in regions of the substrate displaying CD63, indicating specific binding of ACE2 to S–liposomes. Error bars represent standard deviation; *n* = 25 squares. ****: *p* < 0.0001, unpaired two‐tailed *t*‐test.

To show our ability to use DNA‐directed patterning to compare different liposomes featuring distinct surface markers in addition to spike, we fabricated two types of liposomes, one displaying the S trimer and another displaying the second extracellular domain of CD63. We fabricated the S–liposomes and CD63–liposomes to include DiO and DiI dyes, respectively, and tagged them with either Gʹ (S–liposomes) or Aʹ (CD63–liposomes) cholesterol–oligonucleotides. We next flowed a mixture of the tagged liposome types across a substrate featuring arrays with alternating rows of G and A oligonucleotides (**Figure** **4**A,B). The specificity of Watson–Crick base pairing allowed for quick, specific, and simultaneous separation of the tagged liposomes to distinct patterns. After patterning, the introduction of biotinylated ACE2 followed by Cy5–streptavidin allowed us to measure relative ACE2 binding, which occurred only in the presence of S–liposomes and not in the presence of CD63–liposomes (*p* < 0.0001, Figure 4C). Thus, DNA‐directed patterning is a valuable tool to segregate distinct lipid nanoparticles to specific regions of a substrate in order to image selective receptor–ligand binding with a quick and direct fluorescent readout.

**Figure 5:**
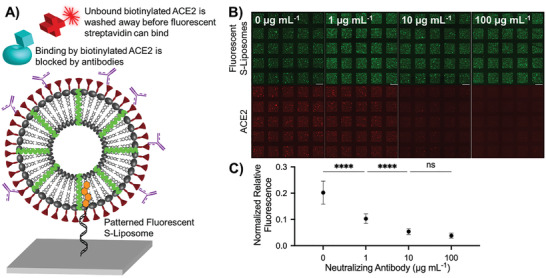
Visualizing and quantifying neutralization activity using DNA‐directed patterning. A) Mechanism by which neutralizing antibodies prevent both the binding of biotinylated ACE2 to DiO‐labeled S–liposomes and subsequent biotin–fluorescent streptavidin interaction. B) Despite uniform liposome‐associated fluorescence (top), fluorescence associated with spike–biotinylated ACE2–fluorescent streptavidin interaction (bottom) decreases as neutralizing antibody concentration increases. Scale bar = 100 µm. C) Quantified fluorescence resulting from spike–biotinylated ACE2–fluorescent streptavidin interaction. Error bars represent standard deviation; *n* = 25 squares. ns: not significant, ****: *p* < 0.0001, one‐way ANOVA with Tukey's multiple comparisons test.

As neutralizing antibodies are a potential therapeutic to treat COVID‐19 patients^[^ ^27^, ^28^, ^29^, ^30^ ^]^ and are actively being developed,^[^ ^31^ ^]^ we employed DNA‐directed patterning to assess neutralizing antibody performance. Specifically, we patterned fluorescent S–liposomes onto substrates and tested the ability of a commercially available neutralizing antibody^[^ ^32^ ^]^ (clone: AS35) to interfere with ACE2 binding. Using a fluorescent secondary antibody, we first confirmed that the neutralizing antibody could bind to the S protein conjugated to our liposome model, even in the presence of off‐target CD63–liposomes (Figure S5, Supporting Information). We then patterned fluorescent S–liposomes and incubated them with different concentrations (0–100 µg mL^−1^) of neutralizing antibody. Following an incubation with biotinylated ACE2, we quantified the neutralizing activity of the antibody based on the detection of fluorescence associated with Cy5–streptavidin (**Figure** **5**A). As expected and shown in Figure 5B,C, the concentration of neutralizing antibody inversely correlated with Cy5 intensity, which agrees with our cell culture experiments (Figure S4B, Supporting Information) as well as an enzyme‐linked immunosorbent assay (ELISA) analysis we performed using AS35 and liposomes (Figure S6, Supporting Information). Our effective, straightforward assay demonstrates the power of DNA‐directed patterning of lipid nanoparticles as a quick and customizable, high‐throughput validation tool. Moreover, our assay also shows that patterned S–liposomes can be used to identify and screen therapeutics that interfere with the binding of ACE2 to the SARS‐CoV‐2 S protein.

**Figure 6:**
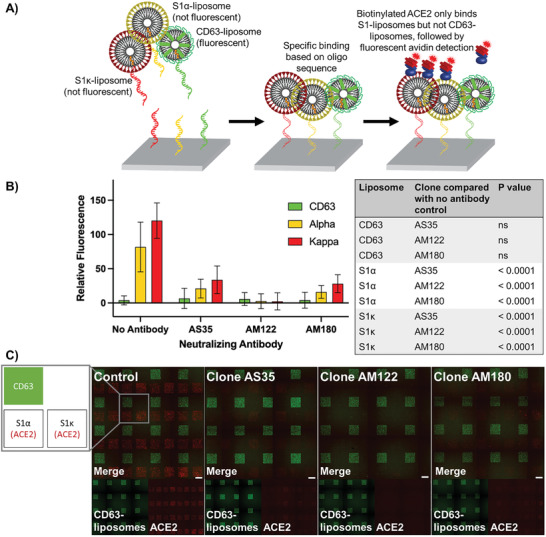
Multiplexed capabilities of DNA‐directed patterning to assess neutralizing antibody performance against multiple SARS‐CoV‐2 variant models. A) Experimental schematic showing DNA‐directed patterning of S1*α*‐, S1*κ*‐, and CD63–liposomes to specific regions, subsequent selective binding of biotinylated ACE2 to spike‐presenting liposomes, and fluorescent streptavidin‐based detection. B) Comparison of ACE2 binding to S1*α*–, S1*κ*–, and CD63–liposomes alone or in the presence of one of three neutralizing antibody clones, AS35, AM122, or AM180. Error bars represent standard deviation; *n* = 16 squares. Two‐way ANOVA with Dunnett's multiple comparisons test. C) Layout and sample images from DNA‐directed patterning assay. Scale bar = 100 µm.

Finally, to highlight the multiplexing abilities of DNA‐directed patterning, we designed a single‐slide assay that simultaneously tested the capability of three neutralizing antibody clones, AS35, AM122, and AM180, to block ACE2 binding to S–liposomes displaying the S1*α* variant subunit or the S1*κ* variant subunit. DiO liposomes displaying CD63 and incorporated with DiO were used as a negative control. By labeling each type of liposome with a different oligonucleotide sequence, we were able to immobilize each liposome variant or CD63–liposome to specific regions of the slide—all in one shot. We could then readout results for each variant based on location, using the DiO CD63–liposome as a visual marker for relative positioning of nonfluorescent liposome variants (**Figure** **6**A,C). We showed significant decrease in ACE2 binding in the presence of all three neutralizing antibody clones, with AM122 displaying the best performance across both variants (Figure 6B). This assay also reflected the performance of a neutralizing antibody against the two variants in a traditional ELISA (Figure S6, Supporting Information). In general, the high‐throughput multiplexing of DNA‐directed patterning allows multiple comparisons of functionality in a single assay.

## 3 Conclusion

In conclusion, we have demonstrated a flexible, easy‐to‐use, high‐throughput method—DNA‐directed patterning—to validate and characterize a lipid‐based nanoparticle, in this case, a model of SARS‐CoV‐2. As we showed, DNA‐directed patterning allows us to pattern S–liposomes for different functional assays, including one that measure binding of ACE2, another that tests the differential binding of relevant proteins to liposomes displaying different surface markers, and one that screens the effectiveness of neutralizing antibodies. Moreover, the flexibility of DNA‐directed patterning of these lipid bilayer nanoparticles enables high‐throughput studies and/or screening capabilities against those displaying different surface proteins or variants of a surface protein, all simultaneously on a single substrate. As we showed, we could use DNA‐directed patterning to design assays that sequester specific liposomes to distinct regions of a substrate for multiplexed assays. In validating S–liposomes, we have also developed a nanoparticle that could be used to optimize safely COVID‐19 diagnostic strategies.^[^ ^33^ ^]^

An attractive feature of our method is that it uses entirely off‐the‐shelf components—oligonucleotides of any 20‐nucleotide sequence can be readily purchased. While we demonstrated the segregation of three different liposome types here using three oligonucleotides with different sequences, the multiplexing capability of this technique, in theory up to 4^20^ oligonucleotide sequences and liposome types, makes possible the analysis of highly heterogeneous solutions of particles. The studies we performed here involved 25 mm × 75 mm glass slides; however, far larger substrates for large‐scale arrays are entirely possible. The spatial resolution and registration we demonstrated are limited by the lithographic technology we employed. Using Mylar masks that are optimal for rapid prototyping, we demonstrated feature sizes as small as 10 µm; however, we could improve this spatial resolution by using a chrome mask or e‐beam lithography. Based on a 10 µm feature size, we can create arrays on the order of 10^8^ different squares on a single microscope slide; this would be equivalent to ≈10^5^ 384‐well plates or 10^6^ 96‐well plates. Finally, our proof‐of‐concept studies were limited by manual pipetting, but the incorporation of automated liquid dispensing and custom microfluidic chambers could enable future high‐throughput screening applications, such as selecting different antibody clones for targeted drug delivery.

Although we have focused on validating a model of SARS‐CoV‐2 in our proof‐of‐concept studies, our method is highly generalizable and could be applied to any number of surface‐modified liposomes. For example, there is particular interest in such particles in the field of drug delivery, as targeted liposomal delivery improves pharmacokinetics while reducing negative effects of therapeutics on nontarget cells.^[^ ^34^ ^]^ Several examples of targeted liposome delivery in anticancer applications include MM302, which contains the highly toxic doxorubicin and targets human epidermal growth factor receptor 2 (HER2)‐overexpressing cells, and SGT35, which targets the transferrin receptor.^[^ ^34^ ^]^ Other tumor‐cell‐targeting strategies include folate as a targeting ligand, anti‐HER2, nucleosome‐specific antibodies, vasoactive intestinal peptide, arginine‐glycine‐aspartate (RGD), hyaluronan, anti‐epidermal growth factor receptor (EGFR), galactosylation, and ligands that bind to chondroitin sulfate.^[^ ^3^ ^]^ Given the high interest in surface‐modified liposomes for applications in anticancer therapies, antifungal treatments, and vaccines and adjuvants, to name only a few, the ability to select rapidly the targeting agent best able to bind a receptor or cellular feature could streamline initial steps in particle design.

DNA‐directed patterning represents an advance over current state‐of‐the‐art technologies used to characterize proteins presented on liposomes (Table S2, Supporting Information). Our platform enables extensive multiplexing with a high degree of flexibility in the assays performed. As the liposomes are tethered to a surface, they can be easily imaged in a single plane, thereby enabling the use of advanced microscopy techniques. Thus, DNA‐directed patterning of liposomes can be widely applied in the efficient formulation and implementation of a wide variety of custom assays for rapid implementation and readout.

## 4 Experimental Section

### Liposome Fabrication

Liposomes were fabricated using protocols adapted from the literature.^[^ ^12^, ^35^, ^36^ ^]^ Briefly, dioleoyl phosphatidyl choline, dioleoyl phosphatidyl ethanolamine, dioleoyl phosphatidyl glycerol acid, and cholesterol (Avanti Polar Lipids) were added in a 4:3:2:7 molar ratio, then dissolved in chloroform. For liposomes that were ultimately labeled with the His‐tagged spike trimer, His‐tagged spike variant, or His‐tagged CD63, 1,2‐dioleoyl‐*sn*‐glycero‐3‐((*N*‐(5‐amino‐1‐carboxypentyl)iminodiacetic acid)succinyl) (nickel salt) (Avanti Polar Lipids) was incorporated at 5 mol%. For fluorescent liposomes, DiO, DiI, or DiD (Invitrogen) were incorporated at 1 mol%. Films were dried using dry nitrogen (N_2_) gas, and residual chloroform was removed during an overnight incubation under vacuum. Films were then rehydrated with Dulbecco's phosphate buffered saline (Thermo Scientific) and incubated at 37 °C for 2 h, after which they were vortexed 3 times for 10 s each and bath sonicated for 30 s. The resulting solution was then incubated overnight at 4 °C. In order to achieve the desired diameter, nanoparticles were extruded first through a 400 nm membrane and then through a 100 nm membrane (Avanti Polar Lipids). Liposomes were stored at 4 °C for up to one week. To label with the SARS‐CoV‐2 spike protein, liposomes were incubated with spike protein (ACROBiosystems, reconstituted in deionized (DI) water in accordance with the manufacturer's protocol, UniProt accession number QHD43416.1; Table S3, Supporting Information) at a ratio of 58.5 w/w lipid:protein for 2 h at room temperature, then stored at 4 °C until use. To label with CD63, liposomes were incubated with CD63 (Sino Biological) at a ratio of 2.6 w/w lipid:protein for 2 h at room temperature, then stored at 4 °C until use. These ratios corresponded to a 1000:1 molar ratio of spike to liposome and a 2000:1 molar ratio of CD63 to liposome. While not saturating, these ratios allowed for high protein labeling without leading to nonspecific patterning. For liposomes to be used in multiplexed experiments, labeling was performed in 0.2% bovine serum albumin (BSA, Sigma‐Aldrich).

### Photolithography‐Based DNA Patterning on Glass Substrates

As previously published,^[^ ^16^ ^]^ aldehyde–silane‐coated glass substrates (Applied Microarrays) were spin‐coated with Shipley 1813 positive photoresist (MicroChem) at 3000 RPM for 30 s and subsequently baked at 100 °C on a hot plate for 1.5 min. The substrates were then exposed to a lithographic mask using UV light (365 nm, power density = 9.5 mW cm^−2^, exposure time = 45 s). After exposure, the substrates were developed in MF‐321 (MicroChem) and rinsed thoroughly with DI 18 MΩ water. Amine‐terminated oligonucleotides (IDT, sequences listed in Table S4 in the Supporting Information) in 50 × 10^−3^ m sodium phosphate buffer were then drop cast onto the exposed regions of the substrates and heated at 85 °C in an oven until the liquid completely evaporated. Reductive amination was completed in 0.25% sodium borohydride (Sigma‐Aldrich) in 1× PBS. Substrates were rinsed with DI water, after which photoresist was stripped with acetone (Gallade Chemical), rinsed with DI water, and then dried with dry N_2_ gas. The process was repeated to introduce additional oligonucleotide sequences. Replica‐molded polydimethylsiloxane (PDMS) flow cells (see below) were “inked” with uncured PDMS, aligned on top of the substrate to enclose the DNA patterns, and cured at 70 °C for 1 h (Figure S7, Supporting Information). Patterned substrates were stored under vacuum until use.

### Replica‐Molded PDMS Chambers

To fabricate PDMS flow cells, soft lithography was used. A negative master was fabricated using photolithography. Specifically, SU‐8 2075 (Microchem) was spin‐coated onto a polished silicon wafer at 1600 RPM for 30 s to achieve a resist thickness of 150 µm. The wafer was soft baked at 95 °C for 30 min and then UV exposed (365 nm, 9.5 mW cm^−2^, 27 s) through a photomask defining the PDMS flow cell. Following UV exposure, the wafer was baked at 65 °C for 5 min, then at 95 °C for 12 min. The wafer was developed in SU‐8 developer (Microchem) for 15 min, rinsed with isopropyl alcohol, and dried with dry N_2_ gas. For PDMS molding of the negative master, a ratio of 10:1 w/w Sylgard 184 prepolymer base to curing agent (Dow Corning) was mixed and degassed. The mixture was then poured over the negative master and cured in an 85 °C oven for at least 4 h. Cured PDMS was removed from the mold using a razor blade and cut to fit within a 4‐chamber slide (Millipore).

### DNA Labeling of Liposomes via Cholesterol Intercalation

“Universal anchor” and “Co‐anchor” strands^[^ ^37^, ^38^ ^]^ (Table S4, Supporting Information) consisting of 41 and 20 nucleotides and 3ʹ and 5ʹ cholesterol tags, respectively, were purchased from IDT. To label different liposomes with different oligonucleotide sequences, the universal anchor was incubated with an adhesion strand comprising a 21‐nucleotide anchor hybridization sequence, a T19 spacer, and a 20‐nucleotide sequence complementary to the oligonucleotide patterned onto the glass substrate.

Cholesterol–universal anchor prehybridized with an adhesion strand was added to a liposome suspension (final concentration: 1 × 10^−6^ m) and incubated for 10 min. Co‐anchor was then added at the same concentration and similarly incubated for 10 min. The liposomes were then ready to use in subsequent patterning assays.

### Fluorescence Imaging Assays

Liposome‐associated cholesterol–oligonucleotide tags were hybridized to complementary oligonucleotides patterned on a glass substrate surface by cycling them through PDMS flow cells 10 times, followed by 3 × 100 µL PBS washing. For experiments with multiple types of liposomes, all liposomes were pooled prior to surface patterning. To verify the presence of spike protein on the liposomes, 9.83 µg mL^−1^ biotinylated ACE2 (ACROBiosystems) in 2% BSA in PBS was cycled through the PDMS flow cells 10 times, then incubated for 1 h at room temperature. After 3 × 100 µL PBS washing, 0.4 µg mL^−1^ Cy5‐labeled streptavidin (Invitrogen) in 2% BSA was cycled through 10 times, then incubated for 45 min at room temperature. After washing with PBS, the substrates were imaged using an ImageXpress Micro (IXM) High‐Content Imaging System (Molecular Devices).

For neutralization assays, prior to ACE2 incubation, different concentrations of anti‐SARS‐CoV‐2 receptor binding domain neutralizing antibody (range: 0–100 µg mL^−1^; Human IgG1, clones AS35, AM122, AM180; ACROBiosystems) in 2% BSA were incubated with the patterned liposomes for 1 h. To visualize binding of the antibody to the liposomes, a fluorescent secondary antibody (mouse anti‐Human IgG1 AlexaFluor 488, Invitrogen) was applied at 1:500 dilution for 45 min at room temperature.

### Live Cell Assay

293T cells engineered to express ACE2^[^ ^25^ ^]^ (see the Supporting Information for cell culture) were seeded at a density of 10^5^ cells mL^−1^ on poly‐l‐lysine (Sigma‐Aldrich)‐coated plates and allowed to culture overnight. Cells were then incubated with liposomes (or, for neutralizing assays, with a mixture of liposomes and 0–10 µg mL^−1^ neutralizing antibodies) and imaged every 30 min using either a MuviCyte Live Cell Imaging System (PerkinElmer) or an IXM. Quantitative measurements were obtained using a SpectraMax M5 Plate Reader (Molecular Devices).

### Statistical Analysis

Statistical analysis was performed with GraphPad Prism v9.1.2 software. Fluorescence intensity was quantified using FIJI. Fluorescence measurements associated with the fluorescent streptavidin that bound to biotinylated ACE2 were normalized to fluorescence associated with the liposomes. Data were presented as mean +/− standard deviation. Statistical tests used in this paper included unpaired two‐sided *t*‐tests and one‐way or two‐way ANOVA with Tukey's multiple comparisons test or Dunnett's multiple comparisons test; specific statistical tests and sample sizes could be found in the appropriate figure captions. *p* < 0.05 was considered statistically significant.

## Conflict of Interest

M Kozminsky, TR Carey, and LL Sohn have filed a patent (PCT/US20/62957) that includes DNA labeling and patterning of S‐liposomes.

## Supporting information

Supporting Information

Supplemental Movie 1

Supplemental Movie 2

Supplemental Movie 3

## Data Availability

The data that support the findings of this study are available from the corresponding author upon reasonable request.
