# Supplementary material for: DNA‐Directed Patterning for Versatile Validation and Characterization of a Lipid‐Based Nanoparticle Model of SARS‐CoV‐2
Source: Adv Sci (Weinh). 2021 Oct 21;8(23):2101166. doi: 10.1002/advs.202101166 (PMC8646752; doi:10.1002/advs.202101166)
Supplement: Supplementary file 1 — Supporting Information [file ADVS-8-2101166-s002.pdf]

WILEY-VCH

© 2021 Wiley-VCH GmbH

ADVANCED SCIENCE
Open Access

Supporting Information

for *Adv. Sci.*, DOI: 10.1002/advs.202101166

DNA-directed patterning for versatile validation and characterization of a lipid-based nanoparticle model of SARS-CoV-2

*Molly Kozminsky, Thomas R. Carey, Lydia L. Sohn**

Supplemental Methods

Cell culture: 293T cells were engineered to express ACE2 (293T+ACE2). They were cultured in D10 media (DMEM, 10% heat inactivated FBS, 1% P/S, 2 mM L-glutamine) and maintained in an incubator at 37°C with 5% CO_2. They tested negative for mycoplasma on August 10, 2020.

Nanosight: Spike-labeled liposomes were diluted 1:2500 in PBS and analyzed using nanoparticle tracking analysis (NTA) on a Nanosight NS300 (Malvern Panalytical). Five technical replicates of 30s each were captured with a syringe pump speed of 50 µL min^{-1} and camera sensitivity set to '13' and were analyzed with detection threshold set to '3' in the Nanosight software.

Enzyme-linked immunosorbant assay (ELISA): anti-SARS-CoV-2 receptor binding domain neutralizing antibody (clone AS35, ACROBiosystems) was adsorbed to the surface of a non-tissue-culture-treated clear polystyrene 96-well plate by incubating 0.1 µg mL^{-1} antibody diluted in 15 mM sodium carbonate + 35 mM sodium bicarbonate buffer at pH 9.6 overnight at 4°C. Wells were blocked with 2% BSA + 0.05% Tween-20, then 10^{10} spike-liposomes suspended in 0.2% BSA in 1X PBS were added to each well. Neutralizing antibody (clone: AS35; ACROBiosystems) diluted in 0.2% BSA + 0.05% Tween-20 in 1X PBS (range: 0-40 µg mL^{-1}) was then added. Next, biotin-ACE2 (ACROBiosystems) diluted to 0.25 µg mL^{-1} in 0.2% BSA + 0.05% Tween-20 in 1X PBS was added. Streptavidin-HRP (Abcam) diluted at 1:20,000 in 0.05% Tween-20 in 1X PBS was subsequently added, followed by 100 µL TMB-Ultra substrate (Thermo Fisher Scientific). An equal volume of 1M sulfuric acid was added after 20 min to quench the reaction and the optical density at 450 nm was measured using a SpectraMax M5 Plate Reader (Molecular Devices). Prior to each addition, wells were rinsed

3x with 0.05% Tween-20 in 1X PBS. Unless otherwise noted, following each addition, the plate was incubated for 1 hr at 37°C.

Image segmentation and analysis methods for uptake rate calculation: Image analysis was performed in two stages: 1) The brightfield channel was segmented using ImageJ version 1.51s to identify areas containing cells; and 2) The fluorescence channel in the areas containing cells was averaged using Python version 3.6.8 and scikit image version 0.17.2 [https://scikit-image.org/].

The brightfield segmentation was performed in ImageJ using a U Net convolutional neural network[1]. First, the brightfield images were adjusted to the automatic brightness and contrast levels in ImageJ. Then, a representative set of 20 images were annotated by hand to label the areas containing cells. These 20 hand-annotated images were used to fine-tune the U Net ready-trained network (available at https://lmb.informatik.uni-freiburg.de/people/ronneber/u-net/) for 2000 iterations. This fine-tuned network was used to segment all brightfield images in the dataset and identify areas containing cells. At least 11 locations were analyzed per well.

The fluorescence data was analyzed in Python using scikit image. First, a binary closing was performed (using a cross-shaped structuring element) to remove small holes in the segmentation mask produced by the U Net. Then, the final mask was applied to the fluorescence channel data. Finally, the average fluorescence per cell area was calculated by summing the pixel values of all masked fluorescence images within each well, and dividing by the total number of masked pixels within each well.

Table S1: Liposome diameter as determined by nanoparticle tracking analysis. Increase in diameter over time and with the addition of oligonucleotides was likely a result of particle fusion and clumping, respectively.

Days post-extrusion	Trimer-labeled	Oligo-labeled	Mean Diameter (nm)	Std Dev (nm)
0	No	No	127.3	31.9
1	Yes	Yes	263.0	109.8
1	Yes	No	190.3	45.1
8	Yes	Yes	263.1	119.5
8	Yes	No	191.3	51.1

Table S2: Comparison of DNA-directed patterning with current technologies.

Technology	Throughput	Flexibility	Multiplexing capability	Functional surface protein assessment capability
Dynamic Light Scattering	Low	Low	No	No
Electron microscopy	Extremely low	High	No	No
Nanoparticle tracking analysis	Low	Low	No	No
X-ray scattering	Medium	High	Yes	No
Enzyme-linked immunosorbent assay	High	High	Yes	Yes
DNA-directed patterning	Extremely high	Extremely high	Yes	Yes

Table S3: Spike proteins. Information obtained from ACROBiosystems. The manufacturer provides SDS-PAGE and bioactivity ELISA data on the website for all three Spike proteins used in this study. MALS and HPLC data on all three products is available upon request from the manufacturer. For product SPN-C52H9, there is additional SDS-PAGE and negative staining electron microscopy data on the website. Information regarding glycosylation pattern can be accessed through UniProt (accession number: QHD43416.1).

Name	Catalog number (ACRO Biosystems)	Amino acids	Mutations	Modifications	Purity (SDS-PAGE)
Trimer	SPN-C52H9	Val 16 - Pro 1213	None	polyhistidine tag at the C-terminus; substitutions for stabilization: F817P, A892P, A899P, A942P, K986P, V987P, R683A, R685A)	>95%
S1α	S1N-C52Hr	Val 16 - Arg 685	HV69-70del, Y144del, N501Y, A570D, D614G, P681H	Polyhistidine tag at the C terminus	>90%
S1κ	S1N-C52Ht	Val 16 - Arg 685	T95I, G142D, E154K, L452R, E484Q, D614G, P681R	Polyhistidine tag at the C terminus	>95%

Table S4: Oligonucleotide sequences. Name indicates distinct strands, with primes corresponding to complementary strands

Purpose	Name	Sequence (5’→3’)
Conjugated to slide surface, 5’ amine	A<br>F<br>G	ACT GAC TGA CTG ACT GAC TG<br>AGA AGA AGA ACG AAG AAG AA<br>AGC CAG AGA GAG AGA GAG AG
Universal Anchor, 3’ cholesterol tag	N/A	TGGAATTCTCGGGTGCCAAGG GTAACGATCCAGCTGTCACT
Universal Co-Anchor, 5’ cholesterol tag	N/A	AGT GAC AGC TGG ATC GTT AC
Adaptor strands to hybridize with Universal Anchor and patterned oligos	A’	CCTTGGCACCCGAGAATTCCA TTTTTTTTTTTTTTTTTTTT CAG TCA GTC AGT CAG TCA GT
	F’	CCTTGGCACCCGAGAATTCCA TTTTTTTTTTTTTTTTTTTT TTC TTC TTC GTT CTT CTT CT
	G’	CCTTGGCACCCGAGAATTCCA TTTTTTTTTTTTTTTTTTTT CTC TCT CTC TCT CTC TGG CT

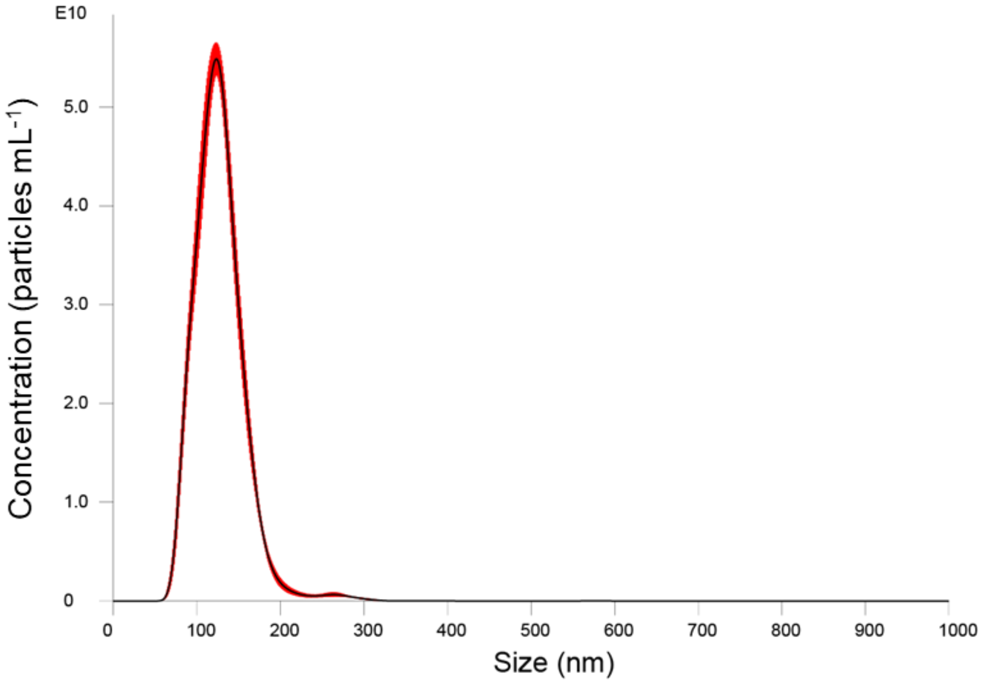

Figure S1: Histogram showing size distribution of S-liposomes as measured using nanoparticle tracking analysis. S-liposomes had a diameter of 127.4 ± 31.8 nm (mean ± standard deviation), and a modal diameter of 123.3 nm. Red shaded region indicates ±1 standard error of the mean.

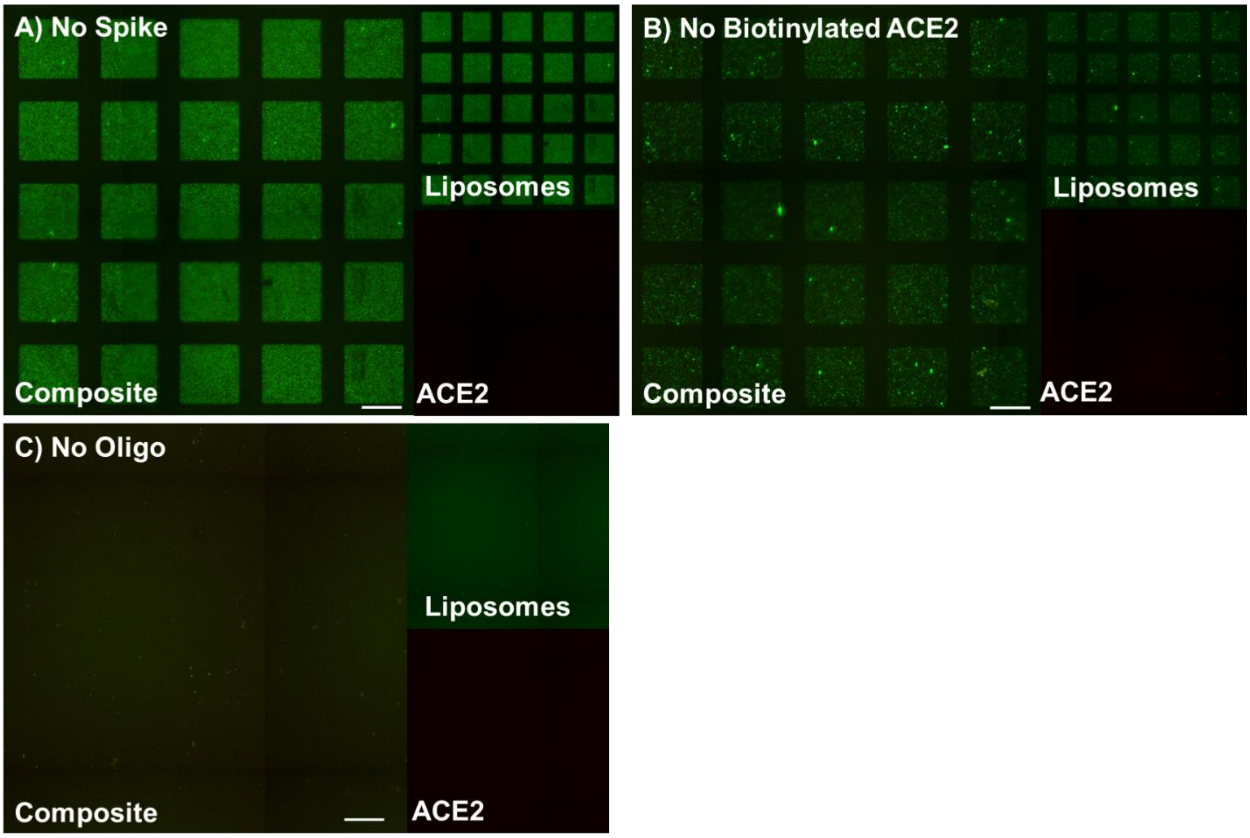

Figure S2: Negative controls for DNA-patterning and binding. (A) DiO-labeled liposomes without spike protein were patterned. The lack of spike prevented the biotinylated ACE2, and subsequently the Cy5-streptavidin, from binding to the liposomes. (B) DiO-labeled S-liposomes were patterned, but the Cy5-streptavidin was added without the biotinylated ACE2, showing minimal nonspecific binding of the Cy5-streptavidin. (C) DiO-labeled S-liposomes without complementary oligo tags were added to a DNA-patterned surface. As they did not pattern, no fluorescence was detected. Scale bar = 100 µm.

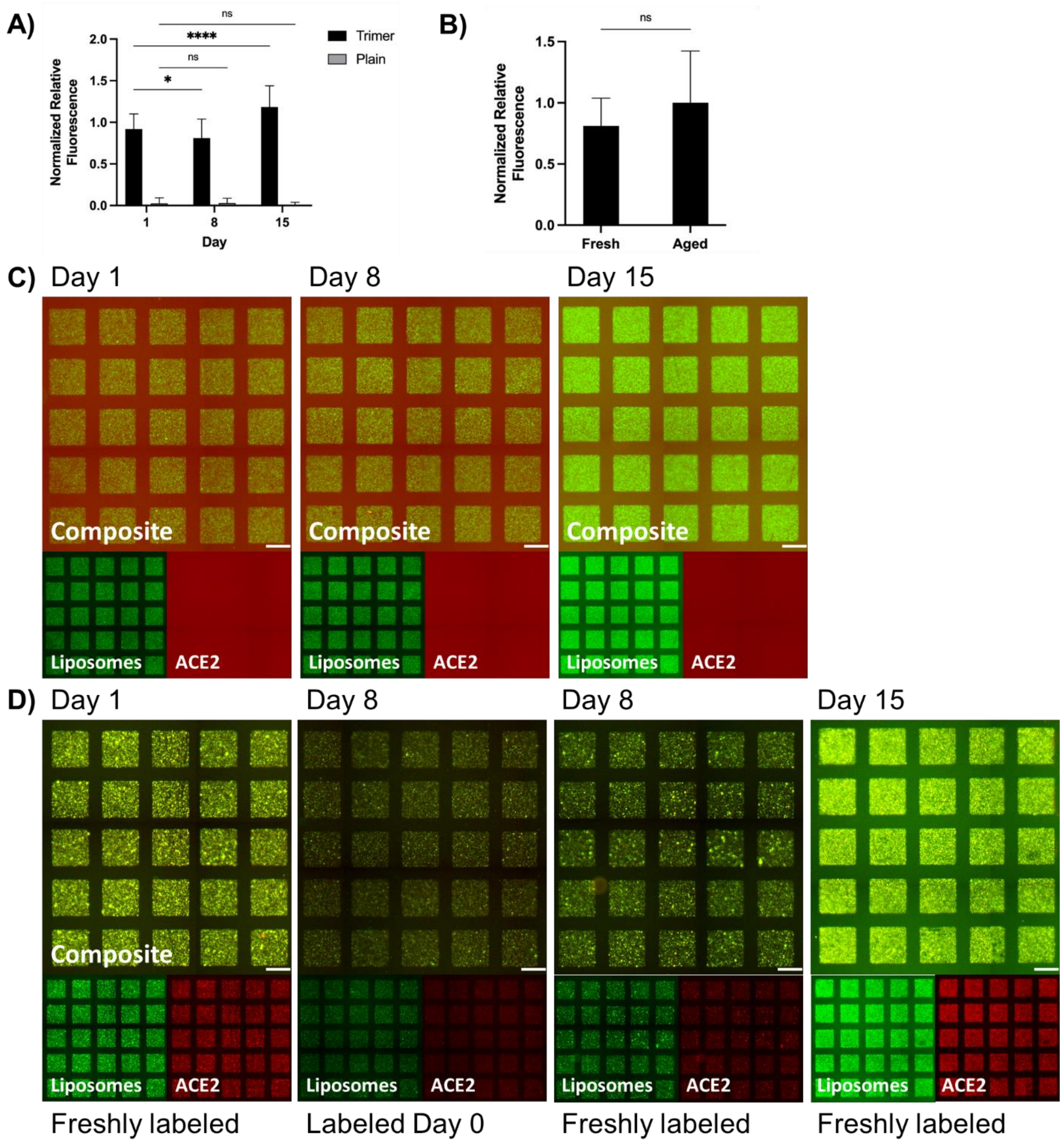

Figure S3: Liposome stability over time. (A) Quantified fluorescence from fluorescent streptavidin binding biotinylated ACE2. There was negligible ACE2 binding to liposomes lacking the spike trimer, while there was some variability in ACE2 binding to S-liposomes based on liposome age, likely a result of degree of clumping and fusion of the older liposomes. This underscores the importance of using liposomes of the same age when performing comparisons, which reflects how experiments were performed in this study. Error bars represent standard deviation; n = 25 squares. ns: not significant, *: p<0.05, ****: p<0.0001, one-way ANOVA with Dunnett's multiple comparsions test. (B) Eight-day-old liposomes labeled on Day 1 and eight-day-old labeled liposomes labeled on Day 8 performed similarly in binding experiments. Error bars represent standard deviation; n = 25 squares. ns: not significant, unpaired two-tailed t-test. (C, D) Plain and S-liposomes, respectively, patterned and assessed for binding with biotinylated ACE2 and fluorescent streptavidin. Brightness/contrast are standardized across all images of the same channel. Scale bar = 100 µm.

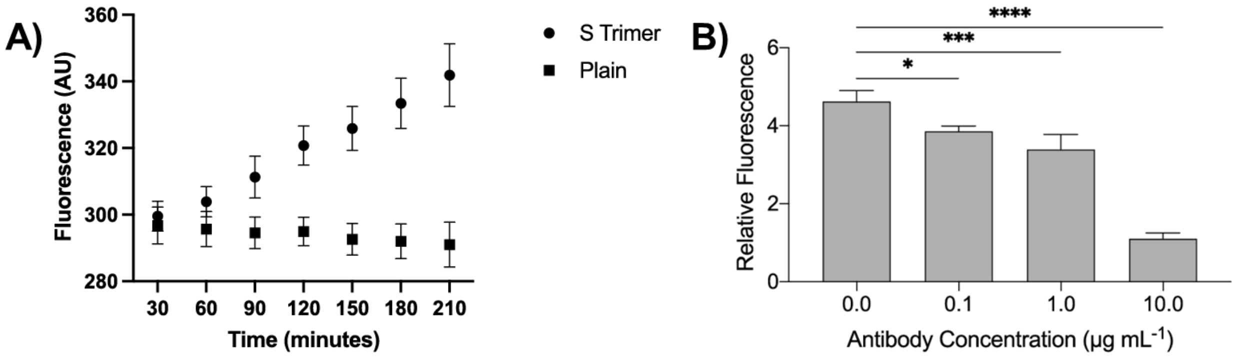

Figure S4: Spike-liposome uptake and antibody neutralization. (A) ACE2-expressing cells (293T+ACE2) were incubated with DiO liposomes featuring either the S protein or the CD63 protein for 3.5 hours, during which they were imaged every 30 minutes. Error bars represent standard deviation; n = 3 wells. (B) S-liposomes were added to 293T+ACE2 cells with neutralizing antibody (clone: AS35; range: 0-10 μg mL^{-1}). Fluorescence intensity was negatively correlated with increasing neutralizing antibody concentration, indicating that the neutralizing antibody inhibited the spike-ACE2 interaction. Unexpectedly, the addition of high concentrations of neutralizing antibody led to lower levels of S-liposome-associated fluorescence than that which occurred in the case of liposomes without spike protein (see Main Text **Figure 3C**). This may be due to the antibody binding to the spike on the S-liposomes and blocking non-specific interactions between these nanoparticles and cells. Error bars represent standard deviation; n = 3 wells for each condition. *: $p<0.05$, ***: $p<0.001$, ****: $p<0.0001$, Dunnett's multiple comparisons test.

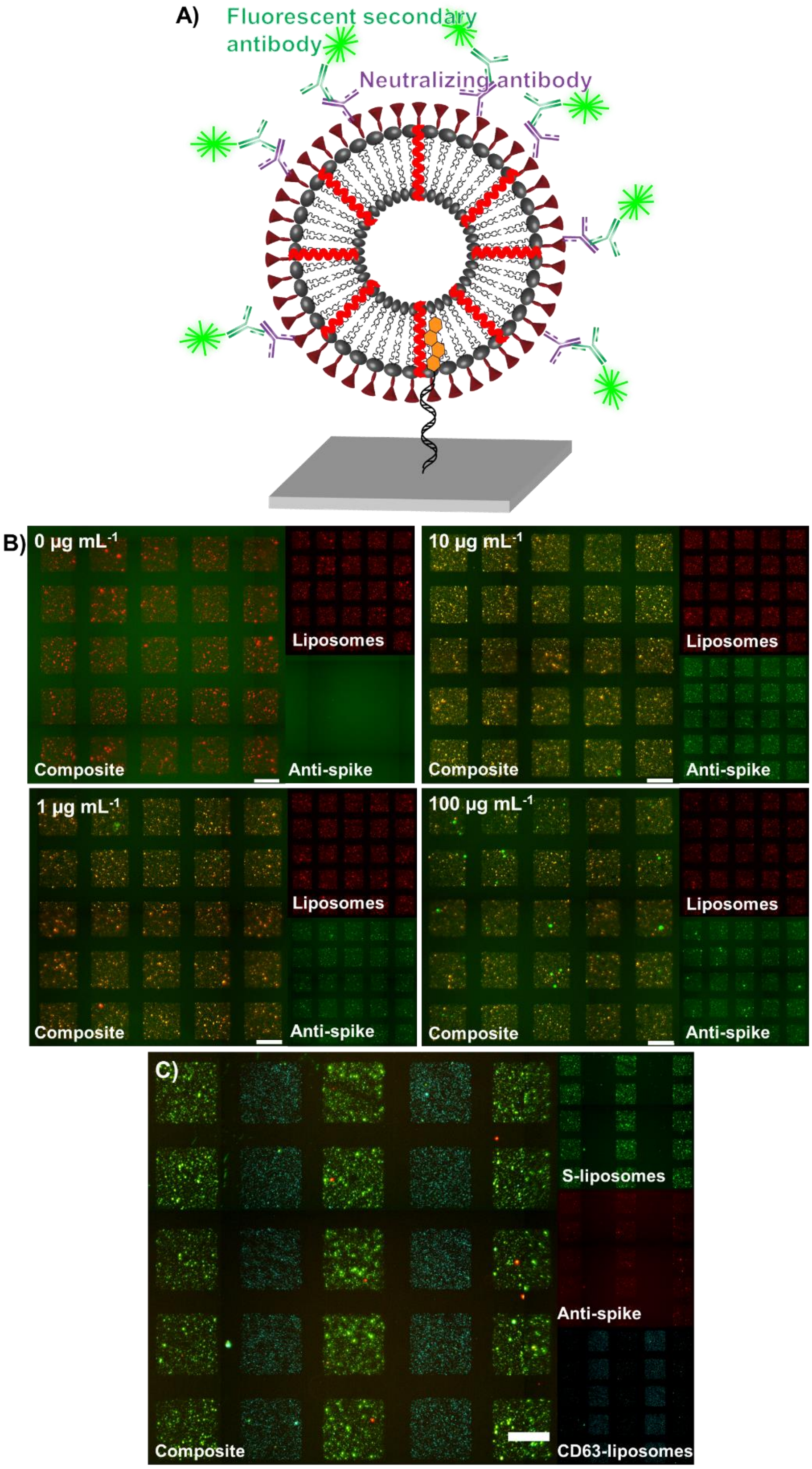

Figure S5: Verifying binding of neutralizing antibodies to S-liposomes. (A) Schematic that shows the detection of spike protein on DiD-labeled liposomes using neutralizing antibody and FITC-labeled secondary antibody. (B) Detection of spike for all non-zero concentrations (range: 0-100 µg mL^{-1}) of neutralizing antibody. (C) Demonstration of specific binding of neutralizing antibody (100 µg mL^{-1}) to S-liposomes in the presence of liposomes presenting off-target protein (i.e. CD63). Scale bar = 100 µm.

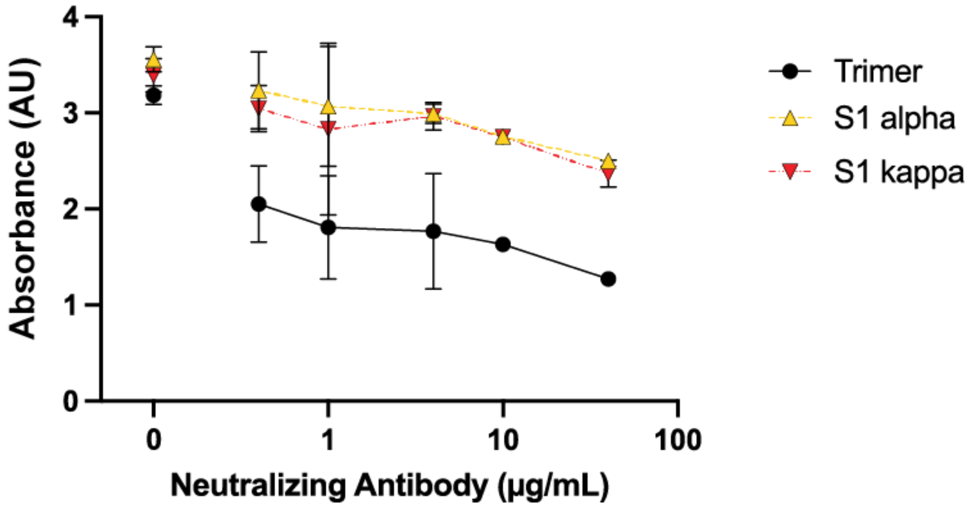

Figure S6: Assessment of S-liposomes-ACE2 binding and its disruption using neutralizing antibody using an enzyme-linked immunosorbant assay. S-liposomes featuring the S trimer or a subunit variant were immobilized on the surface of a 96-well plate using a capture antibody and detected using biotin-ACE2 and streptavidin-horseradish peroxidase (colorigenic read-out via 3,3',5,5'-tetramethylbenzidine (TMB) substrate). The addition of neutralizing antibody (clone: AS35) interfered with the ACE2-spike interaction. ACE2-spike binding decreased with increasing neutralizing antibody concentration, reflecting trends observed in the patterning assays (**Figures 4 and 5**). Error bars represent standard deviation; n = 2 wells.

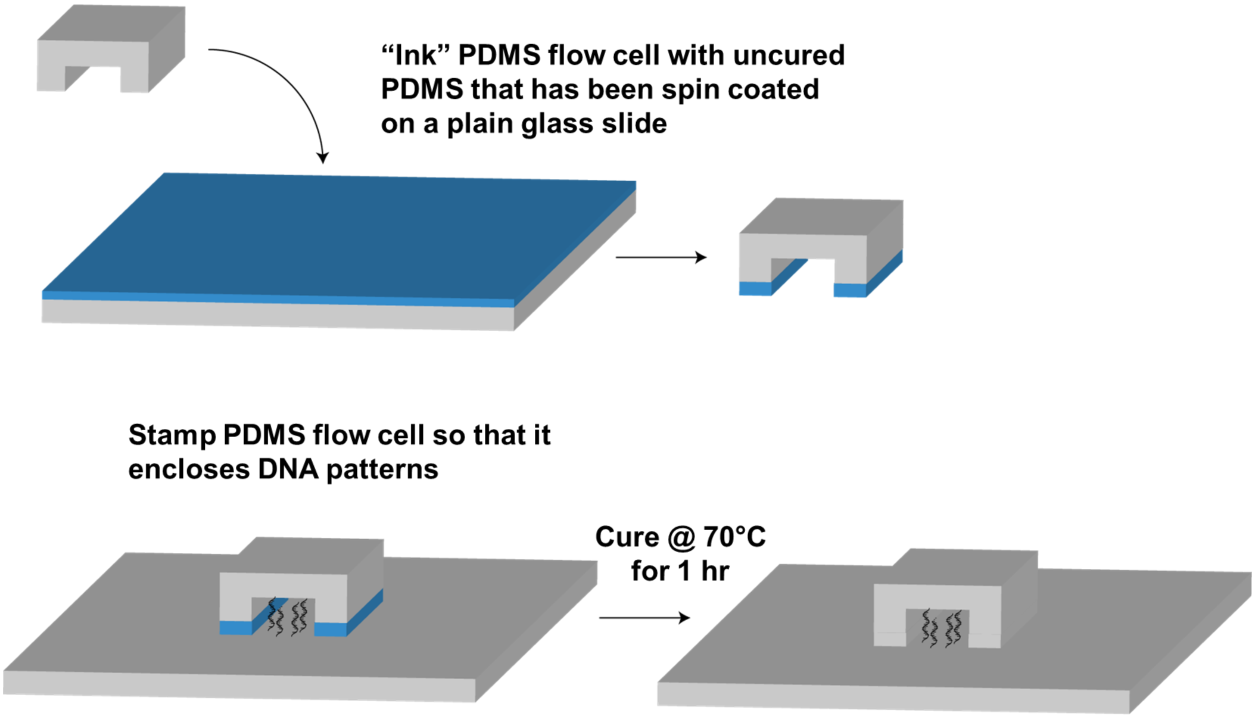

Figure S7: Stamping polydimethylsiloxane (PDMS) flow cells. Pre-cut PDMS flow cells were "inked" with uncured PDMS that had been spin coated on a plain glass slide, then positioned such that they enclosed the DNA patterns. The slide was heated at 70°C for 1 hour to cure the PDMS "ink."

Supplementary Movie 1: Time-lapse images of 293T+ACE2 cells internalizing DiO-labeled S-liposomes. Cells were imaged every 30 minutes for 3.5 hours using a 10x objective on a MuviCyte Live Cell Imaging System.

Supplementary Movie 2: Time-lapse images of 293T+ACE2 cells internalizing DiO-labeled S-liposomes. Cells were imaged every 30 minutes for 4 hours using a 40x objective on an ImageXpress Micro High-Content Imaging System.

Supplementary Movie 3: Time-lapse images of 293T+ACE2 cells internalizing DiO-labeled S-liposomes. Cells were imaged every 30 minutes for 4 hours using a 40x objective on an ImageXpress Micro High-Content Imaging System.

References

[1] O. Ronneberger, P. Fischer, T. Brox, in *Medical Image Computing and Computer-Assisted Intervention – MICCAI 2015* (Eds.: N. Navab, J. Hornegger, W.M. Wells, A.F. Frangi), Springer International Publishing, Cham, **2015**, pp. 234–241.
